# BH3-only蛋白在非小细胞肺癌靶向治疗中的作用及意义

**DOI:** 10.3779/j.issn.1009-3419.2014.11.08

**Published:** 2014-11-20

**Authors:** 巍松 高, 文彦 黄, 凯珊 刘

**Affiliations:** 510632 广州，暨南大学医学院病理系 Department of Pathology, Medical College, Ji'nan University, Guangzhou 510632, China

**Keywords:** 肺肿瘤, BIM, BH3-only蛋白, 靶向治疗, 凋亡, Lung neoplasms, BIM, BH3-only protein, Apoptosis, Target therapy

## Abstract

肺癌死亡率居全球恶性肿瘤死亡之首，非小细胞肺癌是肺癌中最常见的类型。在传统的抗肺癌治疗中，促进细胞凋亡是非小细胞肺癌治疗的一个重要组成部分，但抗肿瘤药物存在毒副作用大和耐药性等问题。因此，寻找新的抗肿瘤药物作用靶点成为非小细胞肺癌治疗的重点之一。BH3-only蛋白在凋亡的启动及凋亡通路的沟通中发挥极其重要的作用。BIM是BH3-only蛋白家族中的核心成员。以BIM为靶点的治疗在非小细胞肺癌的治疗中具有不可取代的作用。本文简单介绍了BCL-2家族和其中的BH3-only促凋亡蛋白，并且阐述了BIM、BH3-only蛋白在非小细胞肺癌靶向治疗中的重要作用。

## Abstract (en)

Lung cancer is the leading cause of cancer deaths throughout the world. Non-small cell lung cancer (NSCLC) is the most common type of lung cancer. In traditional anti-cancer therapy, promotion of apoptosis in NSCLC is an important part of treatment, but anticancer drugs have the toxic side effects, resistance and other problems. Therefore, the search for new targets of anticancer drugs becomes one of the foci in the treatment of NSCLC. The BH3-only protein plays an important role in activation and communication in apoptosis pathways. BIM is the core member in BH3-only protein family. The target at BIM in the treatment of NSCLC has an irreplaceable role. This paper briefly describes the BCL-2 family and BH3-only pro-apoptotic protein, elaborates the important role of BIM and BH3-only protein in targeted therapy of NSCLC.

肺癌是全球发病率和死亡率最高的恶性肿瘤，而非小细胞肺癌（non-small cell lung cancer, NSCLC）是肺癌中最常见的类型。NSCLC分为腺癌、鳞癌和大细胞肺癌等，其中肺鳞癌与吸烟有非常密切的关系。NSCLC是最常见的晚期转移性肿瘤，若不及时治疗，中位存活时间为4个月-5个月，1年生存率小于10%^[1]^。肿瘤发生不仅与细胞的异常增殖和分化有关，也与细胞的异常凋亡有关。在肿瘤形成的过程中，*bim*基因及其相关信号通路在细胞凋亡通路尤其是内源性凋亡通路中有重要的作用，以BIM为靶点的NSCLC靶向治疗具有不可取代的地位。

## 1 BH3-only蛋白概述

### 1.1 细胞凋亡通路及其与BCL-2家族的关系

细胞凋亡信号转导通路主要包括外源性途径即死亡受体凋亡途径和内源性途径即线粒体凋亡途径。内源性线粒体凋亡途径通常是由DNA损伤而激活，通过与促凋亡和抗凋亡BCL-2蛋白家族成员之间的相互作用而调控^[2]^。这两个途径共同激活半胱天冬酶的级联反应，并且介导和执行凋亡细胞蛋白水解反应，调节细胞死亡程序^[3]^。

*Bcl*-*2*基因是目前研究最深入、最广泛的凋亡调控基因之一，存在于线粒体外膜、核膜和内质网膜上。BCL-2蛋白家族可以分为三类：第一类是抑制细胞凋亡蛋白，包括BCL-2、BCL-XL、BCL-W、MCL-1、BCL-B（也称为BCL-2L10）和A1（也称为BCL-2A1）；而第二类是促细胞凋亡蛋白，包括BAX、BAK和BOK（也称为MTD）；第三类是BH3-only蛋白BAD、BIK（也称为BLK或NBK）、BID、HRK（也称为死亡蛋白5，DP5）、BIM（也称为BCL2L11）、BMF、NOXA和PUMA（也称为BBC3）。BH3-only蛋白有一个保守的BH3域，可以结合并调节抗凋亡BCL-2蛋白，促进细胞凋亡^[4]^。

### 1.2 BH3-only蛋白和BIM的作用

BH3-only蛋白仅含BCL-2家族4个同源结构域中的BH3区域，在凋亡的启动及凋亡通路的沟通中发挥着极其重要的作用^[5]^。根据BH3-only蛋白的不同功能可以分为两个亚群：“BH3-only凋亡执行蛋白”，包括BIM、BID等；“BH3-only凋亡感受蛋白”包括NOXA、BMF等^[6]^。BH3-only蛋白是通过线粒体凋亡途径启动细胞凋亡程序的^[7]^。BH3-only蛋白通过抑制BCL-2抗凋亡家族成员，使BAX和BAK释放，引起线粒体外膜透化（mitochondrial outer membrane permeabilization, MOMP），活化半胱天冬酶的级联反应，引起细胞凋亡^[4]^（图 1）。虽然BH3-only蛋白缺乏直接激活BAX和BAK的功能，但是通过与BCL-2家族抗凋亡蛋白结合，间接激活BAX和BAK ^[8]^。

**1:**
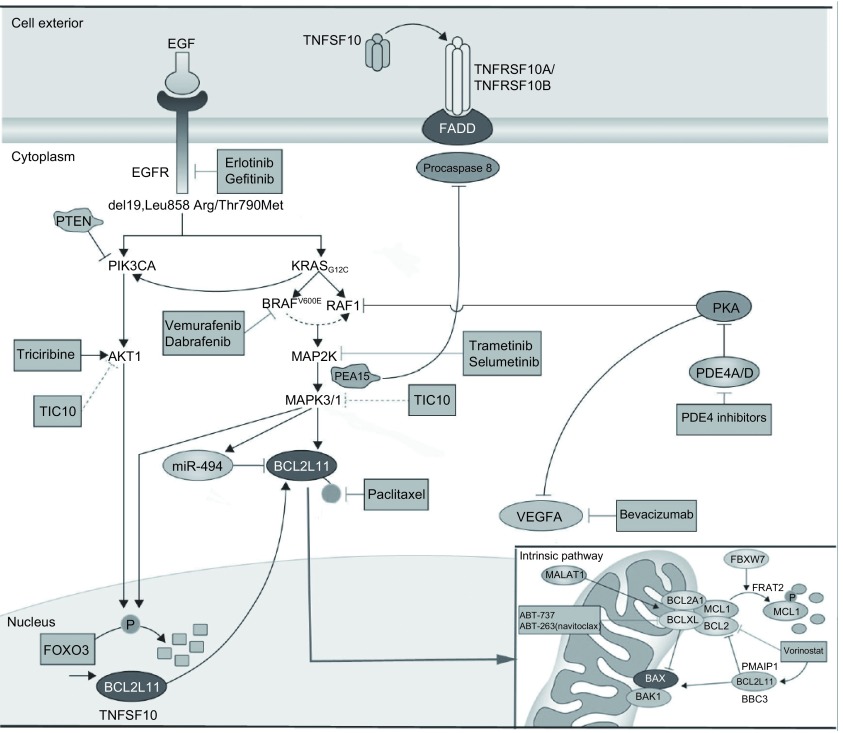
促凋亡蛋白BIM（BCL211）关系图（据文献^[37]^修改） Pro-apoptotic protein BIM (BCL211) diagram (modified according to reference ^[37]^)

BIM在BH3-only蛋白诱导的内源性线粒体凋亡途径中具有核心地位。在人类基因组中，*bim*基因位于7p15.2，编码含129个氨基酸的蛋白质。*Bim*基因编码三个主要的蛋白质亚型：BIMS（BIM short）、BIML（BIM long）和BIMEL（BIM extra long）^[9]^。BIM的所有亚型都含有BH3结构域，结合并抑制BCL-2家族抗凋亡蛋白成员^[10]^。不同的BIM异构体可通过活化BAX蛋白或抑制BCL-2的活性，抑或两者都抑制来调节细胞凋亡。细胞凋亡受阻是NSCLC发生的原因之一。目前NSCLC的治疗以诱导肿瘤细胞凋亡为主。因此，以BIM为靶点的NSCLC治疗，在临床前实验和临床应用中均具有重要意义。

## 2 BIM在NSCLC靶向治疗中的作用

### 2.1 表皮生长因子受体酪氨酸激酶抑制剂（epidermal growth factor receptor tyrosine kinase inhibitor, EGFR-TKI）诱导BIM表达

EGFR在大多数NSCLC患者的癌细胞中都有表达，并参与细胞增殖、抑制细胞凋亡、血管生成、增加肿瘤转移潜能和药物抵抗性^[1]^。抗EGFR特异性的人源化单克隆抗体或小分子TKI能够抑制EGFR^[11]^。EGFR靶向单克隆抗体-西妥昔单抗是唯一的分子靶向剂，已广泛用于评价NSCLC的化疗效果^[12, 13]^。西妥昔单抗加铂类化疗可以作为晚期NSCLC伴有EGFR高度表达患者治疗选择的一个新的一线治疗标准^[14]^。EGFR-TKI——吉非替尼（Gefitinib，易瑞沙）和厄洛替尼（Erlotinib，特罗凯）是第一类用于治疗肺癌的临床靶向药物^[15]^。*EGFR*基因突变存在于大部分对吉非替尼或厄洛替尼敏感的NSCLC中。*EGFR*基因突变与NSCLC对TKI的敏感性增高有关。在*EGFR*突变的NSCLC中，EGFR-TKI通过上调BIM诱导细胞凋亡^[16]^。此外，有数据^[9]^显示BIM的上升程度与凋亡细胞的数量成正比，BIM决定了肺癌细胞对TKIs的凋亡敏感性。从一系列*EGFR*突变的肺癌中评估bim mRNA，Faber等^[17]^证实，bim可作为EGFR-TKIs敏感性的相关标记物。此外，吉非替尼的治疗效果显示，bim表达水平低且有*EGFR*突变的肺癌患者治疗反应差^[17]^。这些结果表明，对于*EGFR*突变的NSCLC尤其是对于突变且TKI抑制剂二次耐药的NSCLC，提高bim的表达水平或激活它下游的靶点很可能是一个有前景的治疗策略^[9]^。

Bim的表达和降解主要受MEK（MAP2K）-ERK（MAPK）信号通路调控，最近研究^[18]^表明MEK-ERK信号通路负性调节bim的表达（图 1）。Bim的表达可增强MEK-ERK信号通路的抑制作用^[19]^。TKI诱导的bim表达上调是对TKI敏感的NSCLC细胞凋亡的重要机制，而TKI作用后bim表达无上调或较少上调很可能是肺癌细胞耐药的重要原因。因此，通过BIM调节细胞凋亡可作为治疗肿瘤的新途径。*Bim*基因多态性的缺失被认为是EGFR-TKIs内在耐药性的新机制^[20]^。这种多态性的缺失使含BH3结构域的BIM蛋白质亚型的表达减少、凋亡诱导失败，导致有*EGFR*突变的NSCLC患者对EGFR-TKI疗效下降、无进展生存期缩短和疾病控制率降低；而且值得注意的是，这种个体突变只发生在东亚人群^[21]^。

### 2.2 Bim表达与抑制肿瘤血管生成的相关性

血管生成是肿瘤生长和转移的必备条件，与肿瘤的发生、发展和转移有着密切关系。血管内皮生长因子（vascular endothelial growth factor, VEGF）及其受体（VEGFR）有促进血管内皮细胞增殖、增加血管通透性、促进血管支持物的生成和抑制肿瘤细胞的凋亡等作用。VEGF系统还可以通过使用特异性酪氨酸激酶抑制剂或单克隆抗体有针对性地抑制VEGFR。无论是药物导致的肿瘤体积缩小或VEGF-A的直接阻滞所引起的VEGF-A缺乏，BCL-2家族BH3-only促凋亡成员bim在血管内皮细胞（endothelial cell, EC）的表达均上调^[22]^。更重要的是，VEGF-A拮抗剂抑制肿瘤生长需要BIM诱导的EC凋亡^[22]^。新的抗癌药物如贝伐单抗（Avastin，基因泰克），是针对VEGF的人源化单克隆抗体，可阻断VEGFR信号，引起EC凋亡和血管退化，已被批准用于一些晚期恶性肿瘤的治疗^[23]^。贝伐单抗、紫杉醇和卡铂（PCB）联合使用的患者，其中位生存期为12.3个月，而且PCB能提高非鳞状NSCLC患者的缓解率（35%），PCB目前已成为晚期非鳞状NSCLC患者的一线标准治疗方案^[24]^。其中，紫杉醇（Paclitaxel）可直接抑制BIM磷酸化和降解，提高BIM蛋白的作用（图 1）。Wang等^[25]^报道，微血管侵袭是NSCLC的肿瘤-淋巴结-转移（tumor-node-metastasis, TNM）分期的重要指标，同时也可能增加患者复发和死亡的风险。因此，肺癌血管生成的研究为肺癌治疗开辟了一条新途径。基于VEGF在肿瘤血管与正常血管表达水平的差异，可以其两者的差异为靶点，阻断新生血管的形成并破坏已形成的血管，从而达到肿瘤治疗的目的。

### 2.3 Bim表达与其转录因子FOXO3（forkhead box protein 3）的关系

FOXO3是FOXO家族的一种转录因子，在肿瘤的发生发展中有抑制功能。PIK3CA/AKT1/FOXO3/BIM信号通路在多种恶性肿瘤中存在，包括NSCLC^[26]^。FOXO3转录因子是PIK3CA/AKT1信号通路的重要信号分子，此通路对FOXO3的表达有一定的负性调控作用，可导致细胞核内FOXO3的磷酸化和降解（图 1）。有研究^[27-29]^表明，在NSCLC、肝癌、胃癌及原发性乳腺癌中均发现FOXO3磷酸化和降解，其中FOXO3的磷酸化和降解还与乳腺癌的低生存率相关。通过调控FOXO3的活性和分布，可促进bim的表达和肿瘤细胞的凋亡，增加NSCLC对抗肿瘤药物的敏感性。紫杉醇和PIK3CA或AKT1抑制剂曲西立滨（Tricirbine）通过抑制AKT1能够减少FOXO3的磷酸化和降解并引起FOXO3核易位，从而导致bim基因的活化和转录。

FOXO3也是药物AZD6244（Selumetinib，司美替尼）的靶点。AZD6244能够增强FOXO3的表达，进一步提高bim表达和诱导细胞凋亡^[30]^。AZD6244也被称为ARRY-142886，为MAP2K小分子抑制剂（图 1）。当AZD6244与多西紫杉醇联合被用于晚期肺癌患者Ⅱ期临床试验时，患者中位生存期相对延长^[37]^。而且，如果敲除FOXO3及其下游的凋亡基因*bim*，AZD6244抑制肿瘤生长的功能减弱^[30]^。Yang等^[30]^观察到对AZD6244耐药的肿瘤，其细胞核的FOXO3受损，同时减弱了FOXO3所导致的转录活性，而且在治疗后FOXO3的靶基因*bim*表达下调。以上证据均提示，PIK3CA/AKT1/FOXO3/BIM信号通路在肿瘤治疗中起着重要作用，为NSCLC多靶点治疗提供了新思路。

## 3 BH3-only蛋白模拟剂与NSCLC靶向治疗

ABT-737是由Abbott公司研发的最成功和最具有开发潜力的BH3模拟化合物^[31]^。BH3模拟剂ABT-737能够与BCL-2、BCL-XL和BCL-W结合，但不能与MCL-1或A1结合^[32]^。在MCL-1被NOXA中和时，ABT-737可引起BAX /BAK依赖的细胞色素C在体外释放^[33]^。有研究^[16]^表明，*bim*基因多态性缺失和*EGFR*突变的NSCLC对吉非替尼所诱导的细胞凋亡有低度敏感性。而BH3-模拟剂ABT-737参与的肿瘤治疗可以极强地抑制BCL-2和BCL-XL，增强TKI诱导的凋亡信号传导和细胞死亡^[16, 34]^。BH3模拟物ABT-737增强了厄洛替尼所导致的细胞凋亡的敏感性^[35]^。因此，EGFR-TKI二次耐药的NSCLC患者联合使用BH3-模拟剂，能够在增强治疗效果的同时减少耐药性和不良反应，这很可能是一种新型的治疗方法。

化学抑制剂ABT-263（navitoclax）是第二代小分子BCL-2蛋白家族口服抑制剂^[36]^，药物机制类似于ABT-737。ABT-263（navitoclax）能够阻止BCL-XL的结合并抑制促凋亡蛋白与MAP2K抑制剂的结合，在许多突变体细胞系中引起细胞凋亡^[37]^。Corcoran等^[38]^在*braf*基因突变的结直肠癌中研究发现，抑制EGFR的活性能阻止*ras*基因的活化，而且BRAF和EGFR抑制剂联合应用使MAPK（mitogen-activated protein kinase）信号活化受阻，增强了抗肿瘤治疗的效果。这提示我们，在*k*-*ras*或*braf*基因突变的NSCLC中，BRAF和EGFR抑制剂联合使用，很可能是一种有前途的治疗策略。MAP2K抑制剂与药物ABT-263合用是对肿瘤细胞有较强杀伤力的治疗，其直接靶点是BIM^[39]^。而且，ABT-263与MEK（MAP2K）抑制剂合用能够在不同组织中诱导*k*-*ras*基因突变的肿瘤细胞凋亡^[39]^。总之，BH3模拟剂能够通过抑制BCL-2家族抗凋亡成员BCL-XL，间接激活BAX，从而引起细胞色素C释放以及随后的半胱天冬酶的级联反应，诱导细胞凋亡。BH3-only蛋白对于肿瘤治疗是至关重要的^[40]^。

## 4 展望

了解细胞凋亡途径和与之相关的分子机制尤其是BIM的作用机制，研究提高细胞凋亡敏感性的方法或直接诱导细胞凋亡对于肿瘤的治疗具有重要意义。在探讨BIM能否成为临床NSCLC防治的参考指标的同时更要思考，是否可以通过人工调节*bim*基因的活性达到对NSCLC的治疗作用。在对*bim*基因进一步研究的基础上，减少抗肿瘤药物的耐药性和不良反应同时不影响机体正常的组织细胞而达到最佳治疗效果是我们的共同目标。然而，与肿瘤凋亡信号通路相关的小分子靶向药物仍存在着多种局限和不足。肺癌易发肿瘤转移^[41]^，目前，在恶性肿瘤的化学治疗中，抗肿瘤药物的毒副作用大以及耐药等不足限制了其进一步的发展，而且肿瘤通常会对起初有效的治疗产生耐药性。因此，寻找新的抗肿瘤药物作用靶点并合成抗肿瘤新药成为肿瘤防治的重点之一。另外，未来的NSCLC靶向治疗将强调针对多个途径不同靶点的组合治疗，以期实现最大的药物疗效并且避开单独用药的不良反应和耐药性。

## References

[1] Blackhall F, Ranson M, Thatcher N (2006). Where next for gefitinib in patients with lung cancer?. Lancet Oncol.

[2] Ashkenazi A, Herbst RS (2008). To kill a tumor cell: the potential of proapoptotic receptor agonists. J Clin Invest.

[3] Ashkenazi A (2008). Targeting the extrinsic apoptosis pathway in cancer. Cytokine Growth Factor Rev.

[4] Youle RJ, Strasser A (2008). The BCL-2 protein family: opposing activities that mediate cell death. Nat Rev Mol Cell Biol.

[5] Plötz M, Eberle J (2014). BH3‐only proteins-possible proapoptotic triggers for melanoma therapy. Exp Dermatol.

[6] Shamas-Din A, Brahmbhatt H, Leber B (2011). BH3-only proteins: Orchestrators of apoptosis. Biochim Biophys Acta.

[7] Happo L, Strasser A, Cory S (2012). BH3-only proteins in apoptosis at a glance. J Cell Sci.

[8] van Delft MF, Huang DC (2006). How the Bcl-2 family of proteins interact to regulate apoptosis. Cell Res.

[9] Costa DB, Halmos B, Kumar A (2007). BIM mediates EGFR tyrosine kinase inhibitor-induced apoptosis in lung cancers with oncogenic *EGFR* mutations. PLoS Med.

[10] Ley R, Ewings KE, Hadfield K (2005). Regulatory phosphorylation of Bim: sorting out the ERK from the JNK. Cell Death Differ.

[11] Potthoff K, Hofheinz R, Hassel J C (2011). Interdisciplinary management of EGFR-inhibitor-induced skin reactions: a German expert opinion. Ann Oncol.

[12] Goldstraw P, Ball D, Jett JR (2011). Non-small-cell lung cancer. Lancet.

[13] Ettinger DS, Akerley W, Bepler G (2010). Non-small cell lung cancer. J Natl Compr Canc Netw.

[14] Pirker R, Pereira JR, Szczesna A (2009). Cetuximab plus chemotherapy in patients with advanced non-small-cell lung cancer (FLEX): an open-label randomised phase Ⅲ trial. Lancet.

[15] Mitsudomi T, Morita S, Yatabe Y (2010). Gefitinib versus cisplatin plus docetaxel in patients with non-small-cell lung cancer harbouring mutations of the epidermal growth factor receptor (WJTOG3405): an open label, randomised phase 3 trial. Lancet Oncol.

[16] Nakagawa T, Takeuchi S, Yamada T (2013). EGFR-TKI resistance due to BIM polymorphism can be circumvented in combination with HDAC inhibition. Cancer Res.

[17] Faber AC, Corcoran RB, Ebi H (2011). BIM expression in treatment-naive cancers predicts responsiveness to kinase inhibitors. Cancer Discov.

[18] Hughes R, Gilley J, Kristiansen M (2011). The MEK-ERK pathway negatively regulates bim expression through the 3'UTR in sympathetic neurons. BMC Neurosci.

[19] Faber AC, Li D, Song Y (2009). Differential induction of apoptosis in HER2 and EGFR addicted cancers following PI3K inhibition. Proc Natl Acad Sci U S A.

[20] Ng KP, Hillmer AM, Chuah CT (2012). A common BIM deletion polymorphism mediates intrinsic resistance and inferior responses to tyrosine kinase inhibitors in cancer. Nat Med.

[b21] 21Zhao M, Zhang Y, Cai W, et al. The Bim deletion polymorphism clinical profile and its relation with tyrosine kinase inhibitor resistance in Chinese patients with non-small cell lung cancer. Cancer, 2014. [Epub ahead of print]

[22] Naik E, O'Reilly LA, Asselin-Labat M-L (2011). Destruction of tumor vasculature and abated tumor growth upon VEGF blockade is driven by proapoptotic protein Bim in endothelial cells. J Exp Med.

[23] Ellis LM, Hicklin DJ (2008). VEGF-targeted therapy: mechanisms of anti-tumour activity. Nat Rev Cancer.

[24] Sandler A, Gray R, Perry MC (2006). Paclitaxel-carboplatin alone or with bevacizumab for non-small-cell lung cancer. N Engl J Med.

[25] Wang J, Wang B, Bi J (2013). Prognostic significance of microvascular invasion and microlymphatic permeation in non-small-cell lung cancer. Eur J Cardiothorac Surg.

[26] Murray ME, Gavile CM, Nair JR (2014). CD28-mediated pro-survival signaling induces chemotherapeutic resistance in multiple myeloma. Blood.

[27] Sunters A, Madureira PA, Pomeranz KM (2006). Paclitaxel-induced nuclear translocation of FOXO3a in breast cancer cells is mediated by c-Jun NH2-terminal kinase and Akt. Cancer Res.

[28] LoPiccolo J, Blumenthal GM, Bernstein WB (2008). Targeting the PI3K/Akt/mTOR pathway: effective combinations and clinical considerations. Drug Resist Updat.

[29] Wali JA, Rondas D, McKenzie MD (2014). The proapoptotic BH3-only proteins Bim and Puma are downstream of endoplasmic reticulum and mitochondrial oxidative stress in pancreatic islets in response to glucotoxicity. Cell Death Dis.

[30] Yang JY, Chang CJ, Xia W (2010). Activation of FOXO3a is sufficient to reverse mitogen-activated protein/extracellular signal-regulated kinase kinase inhibitor chemoresistance in human cancer. Cancer Res.

[31] Elkholi R, Floros KV, Chipuk JE (2011). The role of BH3-only proteins in tumor cell development, signaling, and treatment. Genes Cancer.

[32] Adams JM, Cory S (2007). The Bcl-2 apoptotic switch in cancer development and therapy. Oncogene.

[33] van Delft MF, Wei AH, Mason KD (2006). The BH3 mimetic ABT-737 targets selective Bcl-2 proteins and efficiently induces apoptosis via Bak/Bax if Mcl-1 is neutralized. Cancer Cell.

[34] Soderquist R, Pletnev AA, Danilov AV (2014). The putative BH3 mimetic S1 sensitizes leukemia to ABT-737 by increasing reactive oxygen species, inducing endoplasmic reticulum stress, and upregulating the BH3-only protein NOXA. Apoptosis.

[35] Zheng L, Lin BC, Song ZB (2013). Relationship between *BIM* gene polymorphism and therapeutic efficacy in the retreatment of advanced non-small cell lung cancer with tyrosine kinase inhibitor. Zhongguo Fei Ai Za Zhi.

[36] Tse C, Shoemaker AR, Adickes J (2008). ABT-263: a potent and orally bioavailable Bcl-2 family inhibitor. Cancer Res.

[37] Rosell R, Bivona TG, Karachaliou N (2013). Genetics and biomarkers in personalisation of lung cancer treatment. Lancet.

[38] Corcoran RB, Ebi H, Turke AB (2012). EGFR-mediated reactivation of MAPK signaling contributes to insensitivity of *BRAF*-mutant colorectal cancers to RAF inhibition with vemurafenib. Cancer Discov.

[39] Corcoran RB, Cheng KA, Hata AN (2013). Synthetic lethal interaction of combined BCL-XL and MEK inhibition promotes tumor regressions in KRAS mutant cancer models. Cancer Cell.

[40] Kelly PN, Strasser A (2011). The role of Bcl-2 and its pro-survival relatives in tumourigenesis and cancer therapy. Cell Death Differ.

[41] Jiang R, Ma CH, Zhu ZL (2014). Application of detecting cerebrospinal fluid circulating tumor cells in the diagnosis of meningeal metastasis of non-small cell lung cancer. Zhongguo Xian Dai Shen Jing Ji Bing Za Zhi.

